# An Insight into the Genome of Pathogenic and Non-Pathogenic *Acanthamoeba*

**DOI:** 10.3390/pathogens11121558

**Published:** 2022-12-19

**Authors:** Chayan Sharma, Sumeeta Khurana, Amit Arora, Alka Bhatia, Amit Gupta

**Affiliations:** 1 Department of Medical Parasitology, Postgraduate Institute of Medical Education & Research, Chandigarh 160012, India; 2 Department of Medical Microbiology, Postgraduate Institute of Medical Education & Research, Chandigarh 160012, India; 3 Department of Experimental Medicine & Biotechnology, Postgraduate Institute of Medical Education & Research, Chandigarh 160012, India; 4 Advanced Eye Centre, Postgraduate Institute of Medical Education & Research, Chandigarh 160012, India

**Keywords:** *Acanthamoeba*, granulomatous amoebic encephalitis, hybrid assembly, keratitis, next-generation sequencing

## Abstract

Background: *Acanthamoeba* are amphizoic amoeba majorly responsible for causing *Acanthamoeba* keratitis (AK) and Granulomatous amoebic encephalitis (GAE). Despite its ubiquitous nature, the frequency of infections is not high, probably due to the existence of non-pathogenic isolates. The whole-genome sequencing and an annotated genome assembly can unravel the biological functions and help in identifying probable genes related to pathogenicity. Methods: Illumina and Nanopore sequencing were performed for keratitis, encephalitis, and non-pathogenic environmental isolates. Hybrid assembly was prepared for the AK and GAE isolates, while only the Illumina reads were utilized for a non-pathogenic environmental isolate. Protein coding genes were identified using the GeneMark-ES program and BLASTx module of Diamond used for gene prediction. Additionally, the Kyoto Encyclopedia of Genes and Genomes annotation and cluster of orthologous group’s annotation using RPS-blast against the CDD database was performed. The subsequent data analysis and validation will help identify probable pathogenic genes. Results: The genome assemblies of 9.67, 8.34, and 8.89 GBs were reported for GAE, AK, and non-pathogenic isolate, respectively. KEGG reported 22,946 in GAE, 24,231 in keratitis, and 9367 genes in the environmental isolate. The COG annotation revealed 3232 in GAE, 3403 in keratitis, and 1314 genes in the non-pathogenic isolate. Conclusion: The present study has attempted to generate de novo hybrid genome assemblies of *Acanthamoeba* that would help decode the genome of free-living amoeba and will provide genomic data for a better understanding of virulence-related factors.

## 1. Introduction

*Acanthamoeba* are microscopic, free-living amoebas that are mainly responsible for causing keratitis (AK), granulomatous amoebic encephalitis (GAE), and rarely cutaneous or disseminated disease. Infections due to *Acanthamoeba*, though rare, are often associated with significant mortality or long-term morbidity. Based on 18S rRNA gene sequencing, about 22 *Acanthamoeba* genotypes have been reported (T1-T22) [1,2,3]. The most commonly associated genotype with infection is the T4 genotype, but other genotypes have also been reported, though much less commonly [4,5,6,7,8]. The exact reasons for the greater association of the T4 genotype with human infection are not known. However, few studies point toward the greater transmission ability, greater virulence, and decreased drug sensitivity of the T4 genotype [6,9]. An in-depth genomic analysis might have the potential to differentiate pathogenic isolates from non-pathogenic ones and might also explain the greater prevalence of some genotypes over others. A handful of studies have provided information on the genomes of *Acanthamoeba* species. A study by Clarke et al. reported a genome size of 42.02 Mb for *Acanthamoeba castellanii* and found 15,455 compact intron-rich genes resulting from inter-kingdom lateral gene transfer (LGT) [10]. There is a huge variation in the genome size reported for *Acanthamoeba* species, 42.02 Mb for *Acanthamoeba castellanii*, 49.35 Mb for *Acanthamoeba polyphaga*, 66.43 Mb for *Acanthamoeba triangularis*, and 120.6 Mb for *Acanthamoeba castellanii* ATCC 50370 [10,11,12,13]. However, the exact reason for this variation is not known. In addition, studies in the past explored the proteomic profile of *Acanthamoeba* in various aspects; however, very limited data are available on the genomic aspect.

We report the annotated genome sequence of two pathogenic and one non-pathogenic *Acanthamoeba* isolate duly confirmed for their pathogenicity by in vitro and in vivo animal model studies. The short and long reads raw data have been uploaded to the NCBI Sequence Read Archive (SRA), and the genome project has been deposited at DDBJ/ENA/GenBank. The raw data will be available publicly on the respective databases. 

## 2. Materials and Methods

Three isolates of *Acanthamoeba* were examined in this study. Two of these isolates, obtained from patients who had presented to the Postgraduate Institute of Medical Education and Research, Chandigarh, India, demonstrated pathogenic characteristics in the mouse model. One pathogenic *Acanthamoeba* sp. isolate SK_2022a was obtained from an encephalitis patient, and another isolate, SK_2022b, was obtained from a keratitis patient. Examination of the 18S rRNA gene by a BLAST search indicated that these isolates were most similar to isolate PN14 (GenBank accession # AF333608), assigned to the T11 Sequence type [14,15,16,17]. A non-pathogenic *Acanthamoeba* sp. isolate, SK_2022c, was obtained from a human-made lake of Chandigarh, India, and was confirmed as non-pathogenic in the mouse model by its inability to cause keratitis or encephalitis infections. The 18S rRNA gene sequence of SK_2022c was found to be most similar to *Acanthamoeba* sp. isolate KA/E23 (GenBank accession # EF140625), which has been assigned to the T4 Sequence type, within subtype T4C [14,15,17,18]. 

*Acanthamoeba* keratitis was established in the mouse model by using Parafilm as an alternative to contact lenses. The small lens-like pieces were prepared from Parafilm using a skin biopsy punch, followed by their impregnation with *Acanthamoeba* suspension. The mouse eye was scratched using a surgical blade, challenged with *Acanthamoeba* by using an *Acanthamoeba*-laced Parafilm-lens and tarsorrhaphy was performed [19]. The procedure described previously was used for inducing amoebic encephalitis in the mouse model with slight modifications [20,21]. For this, the mice were treated with two doses of cyclophosphamide (150mg/kg) administered on alternate days. The immunosuppressed mice were then anesthetized and challenged with 10^3^ *Acanthamoeba* trophozoites intranasally. The mice were monitored daily for 21 days for the clinical features of encephalitis that included in-coordination, ruffled hair, and lethargy, and mice presenting with severe clinical features were sacrificed earlier. The mice were sacrificed, and the brain, lung, spleen, and liver were cultured on NNA plates, stored in 10% formalin for histopathology, and used for PCR-based detection of *Acanthamoeba* species [20,21].

### 2.1. Extraction and Sequencing of DNA

The genomic DNA from the *Acanthamoeba* sp. isolates was extracted using Qiagen DNeasy Blood and Tissue kit (Qiagen India Pvt. Ltd., New Delhi, India). The concentration and purity of the genomic DNA were quantified using the Nanodrop Spectrophotometer (Thermo Scientific 2000, Massachusetts, USA) and Qubit dsDNA HS assay kit (Cat#Q32854, Thermo Fischer Scientific, Massachusetts, USA). The integrity of the DNA was observed by agarose gel electrophoresis and the DNA was processed for Illumina and Oxford Nanopore sequencing.

The library preparation was carried out at Genotypic Technology’s NGS facility (Genotypic Technology Pvt Ltd., Bengaluru, Karnataka, India) by an Illumina-compatible NEXTflex Rapid DNA sequencing Bundle (BIOO Scientific, Austin, U.S.A.) library preparation kit, by following the manufacturer’s instructions. Quantification of the libraries was assessed using a Qubit fluorometer (Thermo Fisher Scientific, Massachusetts, USA), and the fragment size distributions were analyzed on Agilent 2200 TapeStation, Germany. The samples were paired-end sequenced on Illumina HiSeq X sequencer (Illumina, San Diego, USA) for 150 cycles. Upon completion, the data were demultiplexed using bcl2fastq software v2.20, and Fast Q files were generated based on the unique dual barcode sequences. The sequencing quality was assessed using Fast QC v0.11.8 software. The adapter sequences were trimmed, and bases above Q30 were considered, and low-quality bases were filtered off during read pre-processing and used for downstream analysis.

For the Oxford Nanopore Technologies, a total of 600 ng of purified DNA (as quantified by Qubit) from the sample was end-repaired (NEBnext ultra II end repair kit; NEB#E7546L, New England Biolabs, Ipswich, MA, USA) and cleaned up with 1x AmPure beads (Beckmann Coulter, Pasadena, CA, USA). Native barcode ligation was performed with NEB blunt/TA ligase (NEB#M0367L, New England Biolabs, Ipswich, MA, USA) and cleaned with 1x AmPure beads. Qubit-quantified barcode ligated DNA sample was adapter-ligated (AMII) for 10 min at 20 °C using NEBnext Quick Ligation Module (New England Biolabs, Massachusetts, USA). The library mix was cleaned up using 0.4X AmPure beads (Beckmann Coulter, Pasadena, CA, USA), and the library was eluted in 15 µL of elution buffer and used for sequencing on SpotON flowcell (FLO-MIN106). Sequencing was performed on GridION X5 (Oxford Nanopore Technologies, Oxford, UK) using SpotON flow cell (R9.4) in a 48-hr sequencing protocol on GridION Release 19.06.9. Nanopore raw reads (‘fast5′ format) were base called (‘fastq5′ format) using a live base calling algorithm (MinKNOW 18.3.3).

### 2.2. Illumina Genome Assembly

All the low-quality Illumina reads were removed using fastp [22]. Kraken2 [23] was used to detect any contamination. Megahit v1.1.3 assembler [24] was used to assemble the reads into contigs. The assembly quality was checked by mapping the reads back onto the assembled contigs using bowtie2 [25]. The assembly completeness was checked using Busco2 with the eudicot_odb10 database [26]. 

### 2.3. Hybrid Genome Assembly

The low-quality Illumina reads were removed using fastp [22]. The quality-trimmed Illumina reads were further checked for the presence of other organisms using Kraken2 [23]. The final reads with the Nanopore long reads were assembled to contigs using Masurca vs. 3.4.2 [27]. The contigs shorter than 500 bp were removed from the assembly. The assembly quality was checked by mapping the reads back onto the assembled contigs using bowtie2 [25]. The genome completeness was checked using BUSCO with the eudicot_odb10 database, which provides a quantitative assessment of the completeness of the expected gene content of a genome assembly [26]. 

### 2.4. Genome Annotation

The protein-coding genes were identified using the GeneMark-ES program [28]. Genes predicted by GeneMark-ES were annotated using the BLASTx module of Diamond [29] against the Uniref100 database. KEGG (Kyoto Encyclopedia of Genes and Genomes) annotations were fetched using KEGG Automatic Annotation Server (KAAS) [30], while COG (clusters of orthologous genes) annotations were performed using RPS-blast against the CDD database using an e-value of 0.001 [31].

## 3. Results

Three *Acanthamoeba* sp. isolates (encephalitis, keratitis, and non-pathogenic isolate) were selected for Illumina and Oxford Nanopore sequencing. All three isolates were confirmed by animal model experiments for their pathogenicity for causing keratitis or encephalitis (data not shown). 

The Illumina sequence reads of the three *Acanthamoeba* sp. isolates were found optimal for downstream analysis. However, the Oxford Nanopore data for the non-pathogenic *Acanthamoeba* sp. isolate was sub-optimal for data analysis, hence; a hybrid assembly was prepared for the two pathogenic isolates (SK_2022a (GAE isolate) and SK_2022b (keratitis isolate)), and only Illumina reads were used for the downstream analysis of the non-pathogenic isolate, SK_2022c (environmental isolate). 

The data was checked for base call quality distribution, percentage bases above Q20, Q30, %GC, and sequencing adapter contamination. There were about 64,461,292 Illumina reads for SK_2022a and 55,566,470 for SK_2022b. The Oxford Nanopore output data accounted for a total length of 2,724,949,085 in the case of SK_2022a and 3,420,889,045 for SK_2022b that was ultimately assembled into 51 MB and 54 MB hybrid genomes for the two pathogenic isolates, respectively. The guanine-cytosine (GC %) content was found to be 56.73% and 57.1% for SK_2022a and SK_2022b, respectively. The details of the genome characteristics and assembly statistics are provided in Table 1 and Table 2. 

The final Illumina reads of the non-pathogenic isolate, SK_2022c, were assembled to contigs using Megahit v1.1.3 assembler. The Illumina sequencing generated 59,234,380 reads accounting for a genome of 22MB with a GC% of 57.91%. 

### Functional Annotation

The prediction of protein-coding genes was performed using the GeneMark-ES program [28]. Genes predicted by GeneMark-ES were annotated using the BLASTx module of Diamond [29] against the Uniref100 database. We found around 65491, 65536, and 65536 genes for SK_2022a, SK_2022b, and SK_2022c, respectively. Among these, the numbers of *Acanthamoeba*-specific genes in SK_2022a, SK_2022b, and SK_2022c were 2934, 2739, and 2306 (Supplementary Table S1).

The gene prediction algorithm GeneMark-ES program generates coordinates of the protein-coding genes predicted in the input genomic sequence. KEGG (Kyoto Encyclopedia of Genes and Genomes) annotations were fetched using KEGG Automatic Annotation Server (KAAS) [30], while COG annotations were performed using RPS-blast against the CDD database using an e-value of 0.001 [31]. 

**Figure 1:**
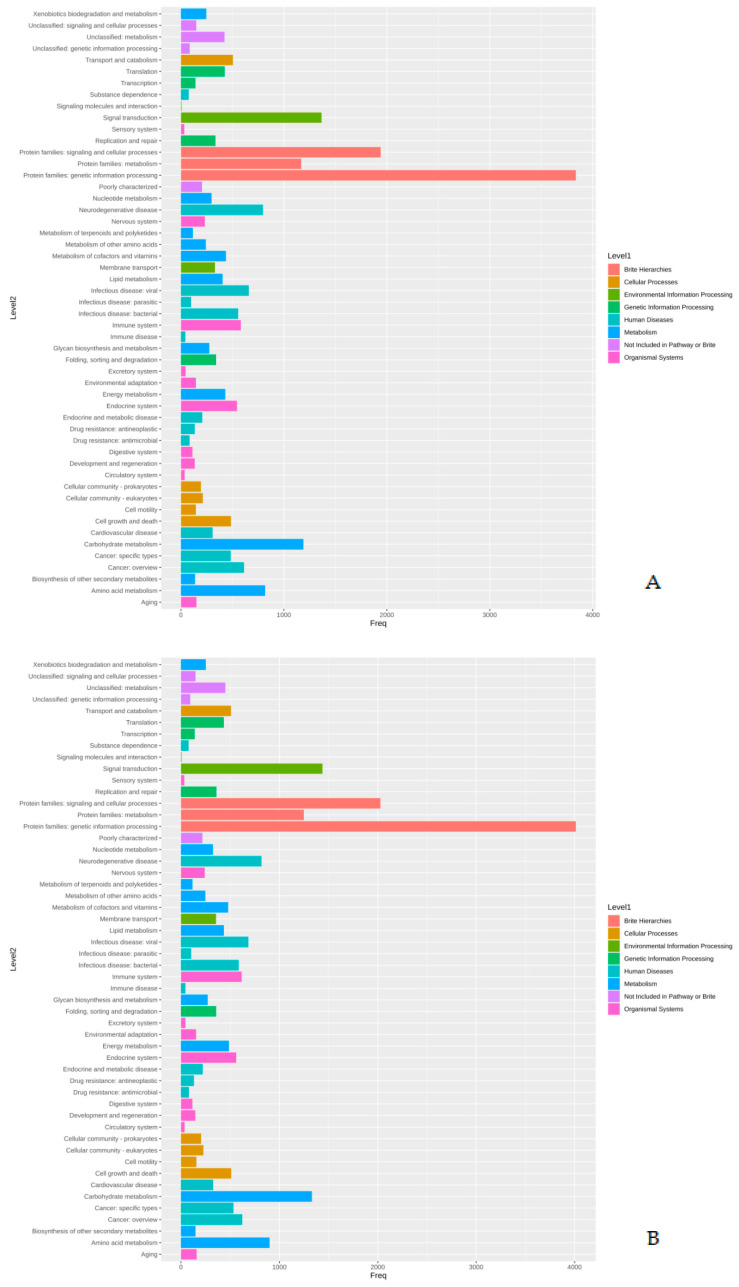

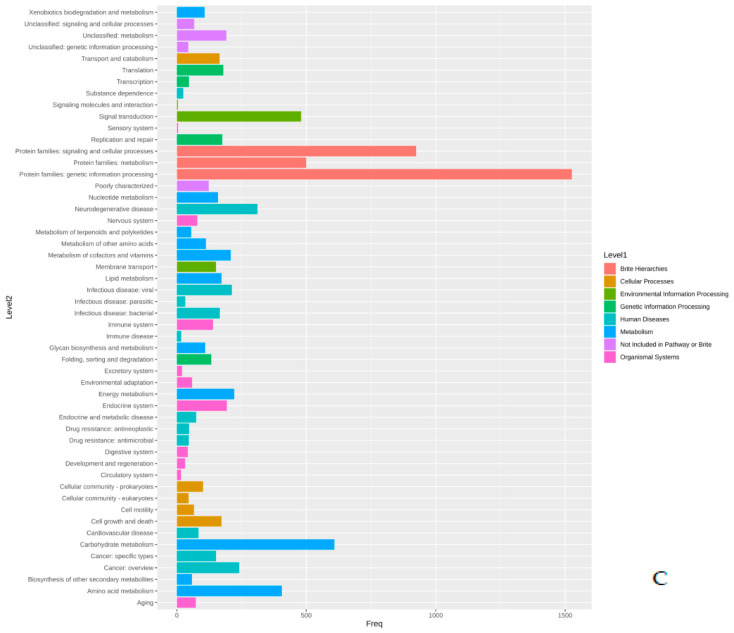
(**A**–**C**). KEGG plots for the three *Acanthamoeba* isolates, (**A**), SK_2022a, (**B**), SK_2022b, (**C**), SK_2022c. SK_2022a, GAE isolate; SK_2022b, keratitis isolate; SK_2022c, non-pathogenic environmental isolate.

**Figure 2:**
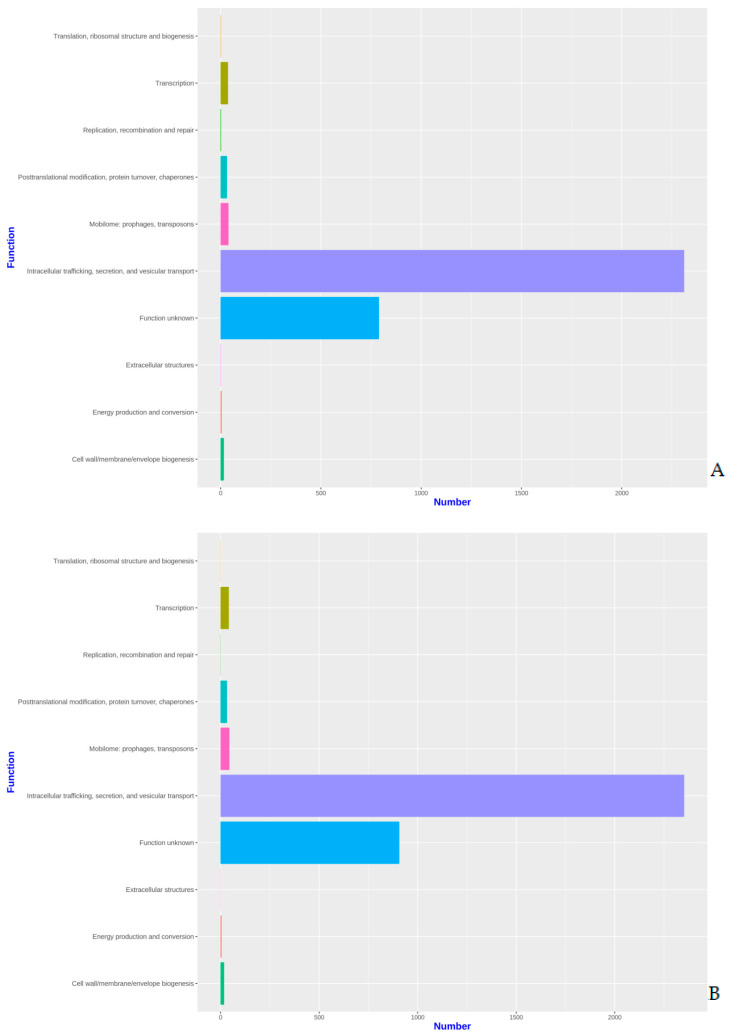

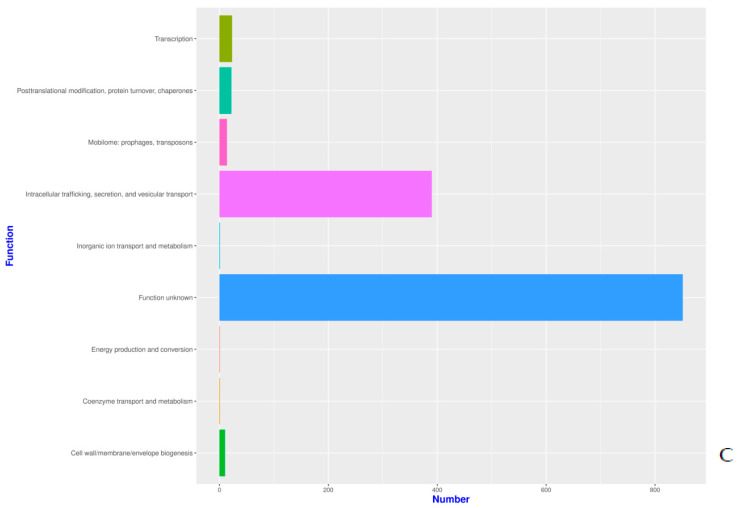
(**A**–**C**). Functional COG plots for the three *Acanthamoeba* isolates, (**A**), SK_2022_a; (**B**), SK_2022b; (**C**). SK_2022c. SK_2022a, GAE isolate; SK_2022b, keratitis isolate; SK_2022c, non-pathogenic environmental isolate.

The proteins identified in the three isolates were subjected to enrichment analysis by the KEGG annotation that designated 5590, 5650, and 2884 functional attributes to SK_2022a, SK_2022b, and SK_2022c, respectively (Supplementary Table S2). In the two pathogenic isolates, about 25.84% of proteins in SK_2022a and 26.10% in SK_2022b were implicated in the metabolism, with the majority of them being involved in carbohydrate metabolism (Figure 1A,B). Additionally, 763, 634, and 599 proteins in SK_2022a and 752, 637, and 595 proteins in SK_2022b were found mapping with genetic information processing, environmental information processing, and cellular processes, respectively. In the non-pathogenic isolate, SK_2022c, 30.23% of proteins were involved in metabolism (Figure 1C), and around 326, 348, and 281 proteins were found mapped with genetic information processing, environmental information processing, and cellular processes, respectively. Based on the COG annotation, 3232, 3403, and 1314 categories of proteins in isolates, SK_2022a, SK_2022b, and SK_2022c, respectively, were mapped to be involved in intracellular trafficking, secretion, vesicular transport function, etc. (Supplementary Table S3). However, about 23.36%, 25.50%, and 62.70% of proteins were assigned to the category of unknown functions in SK_2022a, SK_2022b, and SK_2022c (Figure 2A–C).

**Figure 3:**
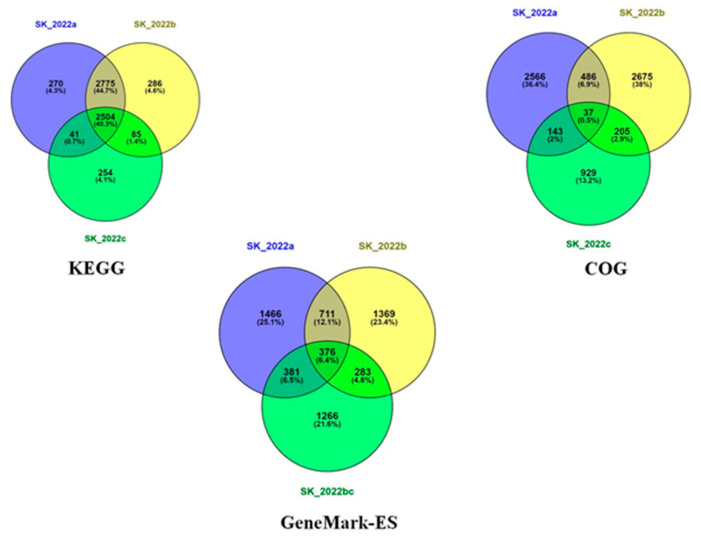
Venn diagram representing GeneMark-ES, Kyoto Encyclopedia of Genes and Genomes (KEGG), and clusters of orthologous genes (COG) data in the three *Acanthamoeba* sp. isolates SK_2022a, GAE isolate, SK_2022b, keratitis isolate, SK_2022c, non-pathogenic environmental isolate.

The genes identified using the GeneMark-ES programs for the three isolates were analyzed using Venny [32]. There were about 711 genes exclusively present in the two pathogenic isolates and absent in the non-pathogenic isolate (Figure 3). Among these 711 genes identified in the two pathogenic isolates, we found genes related to the cytoskeleton (actin-related protein), and lipases (phospholipase and lysophospholipase) that are reportedly involved in host cell lysis. In addition, genes encoding for different peptidases were identified that aid the process of host invasion and oxidoreductase, an important antioxidant defense enzyme (Supplementary Table S1). 

## 4. Discussion

In our study, we attempted to generate hybrid assemblies for the two pathogenic *Acanthamoeba* sp. (T11 Sequence type) isolates and used Illumina reads for assembling the genome of the third non-pathogenic *Acanthamoeba* sp. (T4 Sequence type) isolate. We found approximate genome sizes of 51.3 Mb, 54.7 Mb, and 22.9 Mb for the GAE, keratitis, and environmental isolates, respectively. A total of fourteen draft genome sequences of *Acanthamoeba* species are available publicly on the National Center for Biotechnology Information [13]. The reports in the past have suggested larger genomes for *A. castellanii* ATCC 50370 (120.6 Mb) and *A. polyphaga* ATCC 30872 (115.3 Mb). However, a few studies have reported even smaller genomes for *A. castellanii* Neff (42.02 Mb) and *A. polyphaga* Linc-AP1 (49.35Mb) [11]. In contrast, we observed smaller genomes sizes of 51.3 Mb, 54.7 Mb, and 22.9 Mb for the *Acanthamoeba* sp. isolates. The huge variation in the genome size depends upon the sample source, sequencing technology, and the approach employed for data analysis. The huge genome sizes may be a possible over-exaggeration that could be the result of some errors while assembling the genome.

In our study, the annotation was performed using three approaches; first, the cluster of orthologous genes (COG), followed by the Kyoto Encyclopedia of Genes and Genomes, and GeneMark-ES, which provided information on varied aspects of functional annotation. Using KEGG annotation, proteins in SK_2022a, SK_2022b, and SK_2022c were attributed to metabolism, genetic information processing, environmental information processing, and cellular processes. The cluster of orthologous genes (COG) annotation of SK_2022a, SK_2022b, and SK_2022c reported 3232, 3403, and 1314 proteins responsible for intracellular trafficking, secretion, and vesicular transport function. Additionally, SK_2022a, SK_2022b, and SK_2022c had 23.36%, 25.50%, and 62.70% proteins assigned to the category of unknown functions. The group led by Hasni et al. had reported a large part of genes in the *Acanthamoeba* species genome categorized as “unknown functions” [12]. In addition to COG and KEGG, there are other user-friendly functional genomic databases, such as AmoebaDB, available that could be used for analyzing and exploring gene functions for amoebozoa. AmoebaDB contains genomic data for *Acanthamoeba* and *Entamoeba* species Aurrecoechea 2010 [33]. 

The comparison of *Acanthamoeba*-specific data points among the three isolates generated data regarding the number of genes being shared among any two isolates, or present in all three isolates or present exclusively in any one isolate. This analysis was performed using Venny [32] for COG, KEGG, and the data generated from GeneMark-ES annotation (Figure 3). The GeneMark-ES annotation revealed 2934, 2739, and 2306 genes in SK_2022a, SK_2022b, and SK_2022c, respectively, that were analyzed using Venny, which reported 711 genes in the two pathogenic isolates, SK_2022a and SK_2022b, and absent in the non-pathogenic isolate, SK_2022c. Among these 711 genes, we identified genes encoding for actin-related protein, a cytoskeletal protein responsible for cellular functions associated with cell division and lipases, including phospholipase and lysophospholipase, involved in membrane disruption and cell lysis. In addition to these, different peptidases, including sedolisinlike peptidase, Peptidase, and S8/S53 subfamily protein, were found that help in invading the host. However, this dataset could be subjected to an extensive data analysis and literature survey for identifying probable markers of pathogenicity, which could be further evaluated for their expression profiles in the in vivo *Acanthamoeba* keratitis and encephalitis mouse model. A similar kind of data was reported by Hasni et al. for *A. triangularis* using the Illumina MiSeq technology [12]. However, the use of hybrid assembly followed by de novo genome annotation has the potential to decipher better information. The genome of *Acanthamoeba* strain Neff (ATCC 30010) [10] and *Acanthamoeba triangularis* strain SH621 [12] has been studied in detail previously. The work led by Clark et al. has presented the whole genome assembly of *Acanthamoeba castellanii* as an environmental host and has highlighted the importance of lateral gene transfer in the biology of *Acanthamoeba* [10]. Additionally, the genome sequencing for *Acanthamoeba triangularis* strain SH621 revealed a genome of 66.43 Mb in size that was further evaluated for a better understanding of virulence mechanisms related to the pathogenesis of *Acanthamoeba* keratitis. To the best of our knowledge, one study has recently assembled the genome of *Acanthamoeba* using long and short reads data to provide a better understanding of the chromatin organization during *Legionella pneumophila* infection [34].

The data presented in our study can be used for a comparative analysis between the pathogenic and non-pathogenic *Acanthamoeba* isolates and among the keratitis and encephalitis-causing isolate at the genomic level. Additionally, the identification of the two pathogenic isolates as members of the T11 sequence type is quite significant. To date, there is no whole-genome sequencing (WGS) information reported for the T11 sequence type. Hence these data will be substantial additions to the genomic information on *Acanthamoeba*, and certainly will allow us to understand how various sequence types differ. On the other hand, the identification of the non-pathogenic isolate as a member of sequence type T4C is also significant. As with T11, there is no genomic information reported so far for the T4C sequence type. In fact, it is the only T4 subtype currently without any WGS information. The lack of WGS information for sequence type T4C raises the possibility that an additional explanation for the small size of the genome might be related to a true difference in the genome size within this subtype. This speculation can be answered in light of additional genomic sequences in the future.

The main limitation of this study is the unexplained small genome size of the non-pathogenic isolate. The use of Illumina data alone for assembling the genome of non-pathogenic isolate and the inability to reconstruct a hybrid genome might have resulted in a smaller genome size. However, the paucity of adequate data becomes the limiting factor for providing any such statement that can be explained in light of the appropriate evidence. Nevertheless, the data from pathogenic and non-pathogenic isolates can help identify and characterize pathogenicity-associated genes that would help design better therapeutic approaches. Additionally, genes involved in the mechanism of pathogenesis can be used for designing gene knockout experiments and conducting associated downstream analyses.

## Figures and Tables

**Table 1:** Summary of the *Acanthamoeba* genome characteristics.

Illumina Sequence Data and Quality						
Sample ID	No. of Reads	Data in Gbs	GC %	Read Length	%Q20	%Q30
SK_2022a	64,461,292	9.67	52.5	150	99.26	91.36
SK_2022b	55,566,470	8.34	54.5	150	99.33	90.55
SK_2022c	59,234,380	8.89	56	150	99.32	93.73
Oxford Nanopore sequence data and quality						
Sample ID	No. of sequences	Total length		Average Length	Minimum Length	Maximum Length
SK_2022a	1,059,703	2,724,949,085		2,571.40	30	38,367
SK_2022b	1,240,410	3,420,889,045		2,757.90	28	38,693

SK_2022a, GAE isolate; SK_2022b, keratitis isolate; SK_2022c, non-pathogenic environmental isolate.

**Table 2:** The statistics of the genome assemblies for the *Acanthamoeba* isolates.

Assembly	SK_2022a	SK_2022b	SK_2022c
# contigs (>= 0 bp)	2138	1809	21,308
# contigs (>= 1000 bp)	2138	1809	1634
# contigs (>= 5000 bp)	1728	1666	117
# contigs (>= 10000 bp)	1133	1141	98
# contigs (>= 25000 bp)	508	527	72
# contigs (>= 50000 bp)	218	253	46
Largest contig (bp)	3,411,420	2,013,303	8,88,447
Total length (bp)	51,384,221	54,719,801	22,953,089
GC (%)	56.73	57.1	57.91
N50	49,572	64,210	866
N75	19,912	23,761	634
L50	222	189	3186
Alignment %	79.01%	76.59%	93.01

SK_2022a, GAE isolate; SK_2022b, keratitis isolate; SK_2022c, non-pathogenic environmental isolate.

## Data Availability

The short reads and long reads raw data were uploaded to the NCBI Sequence Read Archive (SRA) and they are available under the accession number SUB10978116 with BioSample numbers SAMN24433775, SAMN24433776, and SAMN24433777 for SK_2022a, SK_2022b, and SK_2022c. This whole genome project has been deposited at DDBJ/ENA/GenBank under the accession JANEZP000000000, JANDJZ000000000, JANDKB000000000.

## Supplementary Materials

The following are available online at https://www.mdpi.com/article/10.3390/pathogens11121558/s1, Table S1: List of *Acanthamoeba* specific genes in SK_2022a (encephalitis isolate), SK_2022b (keratitis isolate), and SK_2022c (non-pathogenic isolate) and a list of the 711 genes identified in the two pathogenic isolates; Table S2: KEGG annotation for SK_2022a (encephalitis isolate), SK_2022b (keratitis isolate), and SK_2022c (non-pathogenic isolate); Table S3: COG annotation for SK_2022a (encephalitis isolate), SK_2022b (keratitis isolate), and SK_2022c (non-pathogenic isolate).
